# A Novel Strategy for the Design of Aurein 1.2 Analogs with Enhanced Bioactivities by Conjunction of Cell-Penetrating Regions

**DOI:** 10.3390/antibiotics12020412

**Published:** 2023-02-19

**Authors:** Fengting Liao, Yuping Chen, Anmei Shu, Xiaoling Chen, Tao Wang, Yangyang Jiang, Chengbang Ma, Mei Zhou, Tianbao Chen, Chris Shaw, Lei Wang

**Affiliations:** 1 Natural Drug Discovery Group, School of Pharmacy, Queen’s University Belfast, Belfast BT9 7BL, Northern Ireland, UK; 2 Department of Basic Medical Science, Jiangsu Vocational College of Medicine, Yancheng 224005, China

**Keywords:** rational peptide design, cell penetrating regions, aurein 1.2, antimicrobial activity, anti-proliferation activity

## Abstract

The rational design modification of membrane-active peptide structures by introducing additional membrane-penetrating regions has become a good strategy for the improvement of action and potency. Aurein 1.2 (GLFDIIKKIAESF-NH_2_) is a multifunctional antimicrobial peptide isolated from the green and golden bell frog, *Litoria aurea*, and the southern bell frog *Litoria raniformis* skin secretions. Its bio-functionality has been widely investigated. However, its lack of a potent action failed to provide aurein 1.2 with a competitive edge for further development as a therapeutic agent for clinical use. Herein, aurein 1.2 was chosen as a template for rational modification to achieve a more potent bio-functionality. KLA-2 (GLFDIIKKLAKLAESF-NH_2_), which a double KLA region inserted into the sequence, presented a 2–16-fold enhancement of antimicrobial activity, a 2–8-fold greater anti-biofilm activity (including biofilm prevention and eradication), and a 7-fold more potent anti-proliferation activity and hence was regarded as the most broad-spectrum active peptide. Additionally, with respect to antimicrobial activity, the IIKK-modified analog, IK-3 (GLFDIIKKIIKKIIKKI-NH_2_), also demonstrated a potent enhancement of activity against various pathogens, exhibiting a 2–8-fold enhanced activity compared to the parent peptide. Moreover, the selectivities of KLA-1 and KLA-2 were enhanced significantly. In conclusion, peptide modification, through the introduction of additional membrane penetrating regions, can increase both the potency and activity spectra of natural template peptides, making them suitable candidates for new drug development.

## 1. Introduction

The inappropriate use of clinical antibiotics, leading to the generation and spread of antibiotic-resistant superbugs, has caused severe problems not only in human medicine but also in veterinary medicine and in agriculture [1]. The rise of antibiotic resistance globally may be largely attributed to the overuse of antibiotics in all biology-related industries [2]. The issue has become much worse as a result of careless global antibiotic sales, poor prescriptive compliance, poor sanitation, and the release of unmetabolized antibiotics or their residues into the environment through feces, manure, and industrial effluents [3]. Approximately 700,000 people die each year from infections with multidrug-resistant microbiological agents [4]. By 2050, the annual mortality rate is anticipated to surpass cancer-related fatalities and reach 10 million if an adequate action plan is not put into place [5]. In the past years, antibiotics use in livestock and poultry has increased at an unprecedented rate worldwide [6]. By 2030, developing countries are projected to increase antibiotic use by 67% [7]. At the same time, the growing global problem of drug resistance has greatly increased the health burden on the world economy [8]. Therefore, alternative antimicrobial therapeutic agents are urgently needed to combat these antibiotic-resistant superbugs. Host defense peptides (HPDs) provide a promising alternative strategy in the treatment of resistant pathogens [9]. Cationic HPDs, normally consisting of 12–50 amino acids with general amphipathicity and cationicity, are small molecules isolated from various sources, including mammals, amphibians, plants, insects, and microorganisms [10,11]. HDPs display broad spectra of antimicrobial activity against both planktonic and sessile (biofilm-forming) bacteria [12]. In addition, some HDPs also show anticancer efficacy [13,14].

Aurein peptides, first isolated from the skin secretion of the green and golden bell frog *Litoria aurea*, are also present in the southern bell frog *Litoria raniformis* and have been found to have broad-spectrum antimicrobial and anticancer efficacies. The aurein peptides can be classified into five discrete groups (aureins-1–5), among which, aurein-1 peptides are regarded as among the shortest of α-helical peptides with antimicrobial and anticancer activities [15]. Aurein 1.2, the most studied peptide in the aurein family, was demonstrated to have broad spectrum bi-functionality against bacterial and cancer cells. However, the activity of this peptide was not remarkable with respect to potency [16].

Short, designed amphipathic peptides are attractive for their potent antimicrobial and anticancer properties and potencies. Additionally, the sequence and design flexibility offer great potential for enhancement of their bio-efficacies [17]. The introduction of short, designed domains with cell membrane penetrating properties into the peptide sequence is one of the most effective modifications in enhancing such bio-activities of peptides [18,19]. 

In this study, two membrane-penetrating domains, KLA and IIKK, were selected in the modification of aurein 1.2, hoping to further increase the antimicrobial and anticancer potencies of the peptide, thus providing this template with a more competitive edge in selection for future development. Herein, peptides IK-1 and IK-2, which contained different repeats of the IIKK region, were designed, together with an N-terminal modified analog, IK-3, to investigate the influence of the pro-apoptosis inducing fragment, IIKK in the middle of the sequence. In addition, the leucine-substituted analog, KLA-1, and the KLA repeat analog, KLA-2, were designed to study the effect of a different cell penetration domain, KLA, on the antimicrobial and anticancer activities of the template peptide. 

## 2. Results

### 2.1. Rational Design and Physiochemical Parameter Acquisition

Aurein 1.2 is a short antimicrobial peptide containing 13 amino acids with an amidated C-terminal. It has been reported that aurein 1.2 has broad-spectrum antimicrobial- and anticancer activities [20]. In this study, the peptide demonstrated anti-proliferation activity against 90% of human cancer cell lines tested. However, its anti-proliferation activity was moderate at concentrations of 10^−4^–10^−5^ M [21,22,23]. In this study, the aim was to further increase the antimicrobial and anti-proliferation activity of aurein 1.2 to obtain a series of potent, broad-spectrum multifunctional peptides. 

Towards the goal of improvement of activity, five analogs of aurein 1.2 were designed and synthesized. Short, designed cell-penetrating peptides have attracted the attention of researchers because of their bactericidal and anti-proliferation functions as well as their structural design flexibility. In Chen’s study, the designed sequence, (IIKK)_3_-NH_2,_ showed potent anti-proliferation activity with a high membrane destruction rate providing an interesting approach to the modification of aurein 1.2 [17]. In the aurein 1.2 sequence, an IIKK region can be found, and by repeating this -IIKK- region once and twice, analogs IK-1 and IK-2 were designed with the hope of achieving a more potent function. By introducing the lysine residue (K) into the sequence, IK-1 and IK-2 gained two and four net positive charges, respectively, compared to aurein 1.2. A point worthy of note was that the hydrophobic moment of these two peptides changed differently. The hydrophobic moment of IK-1 was 0.78 μH, increased compared to aurein 1.2. On the contrary, the hydrophobic moment of IK-2 was reduced after modification.

The -IIKK- sequence with isoleucine at the C-terminus, followed by an amide, seemed to produce a better functional peptide compared to those without isoleucine or terminating with other amino acids [17]. Therefore, the -AESF- region at the C-terminus of IK-2 was removed to make a new analog, IK-3. IK-3 had the highest hydrophobic moment among all the tested peptides.

(KLAKLAK)_2_ is another cell-penetrating antimicrobial peptide that also shows anticancer activity [24,25]. This sequence is always used as a membrane-disrupting (pro-apoptotic) domain in anticancer peptide design [26]. In KLA-1, the isoleucine residue at position nine was substituted by leucine to form a -KLA- region in the sequence. Furthermore, in KLA-2, one more KLA region was introduced in the sequence by introducing a KLAKLA region into the sequence, aiming to attain greater bioactivity. After modification, KLA-1 and KLA-2 both demonstrated a lower hydrophobicity and hydrophobic moment compared to aurein 1.2. All peptide sequences and their physiochemical parameters are listed in Table 1.

### 2.2. Secondary Structure Prediction and Analysis

**Figure 1:**
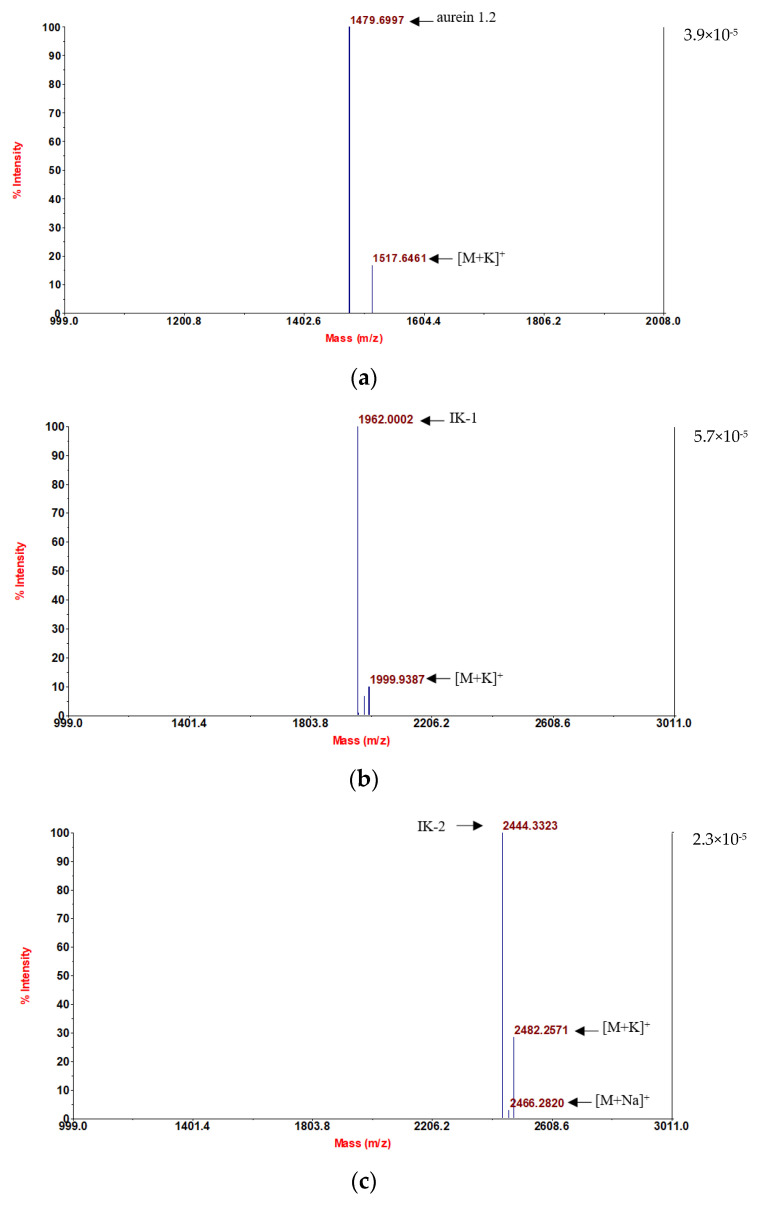

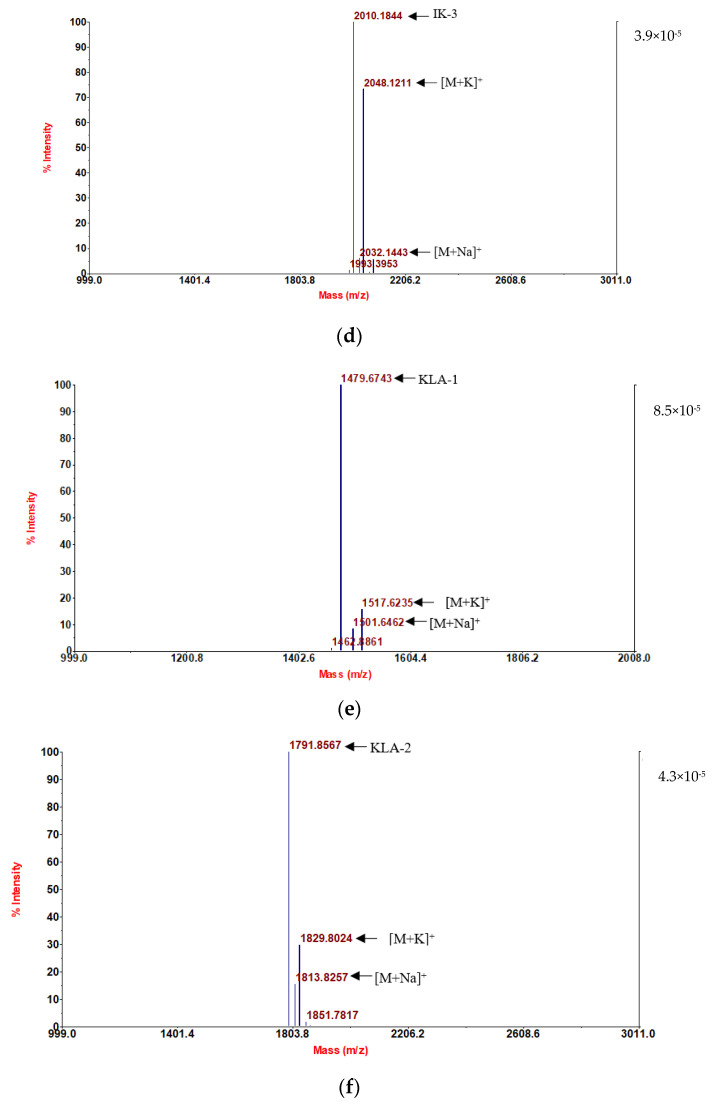
MALDI-TOF MS spectra of the six purified synthetic peptides: (**a**) aurein 1.2; (**b**) IK-1; (**c**) IK-2; (**d**) IK-3; (**e**) KLA-1; and (**f**) KLA-2.

Following convincing confirmation of the sequence, aurein 1.2 and its five analogs were synthesized using solid-phase Fmoc chemistry following the MALDI-TOF MS spectra analysis. The molecular masses of the components are in accordance with the theoretical masses, demonstrating that all six peptides were successfully attained (Figure 1).

**Figure 2:**
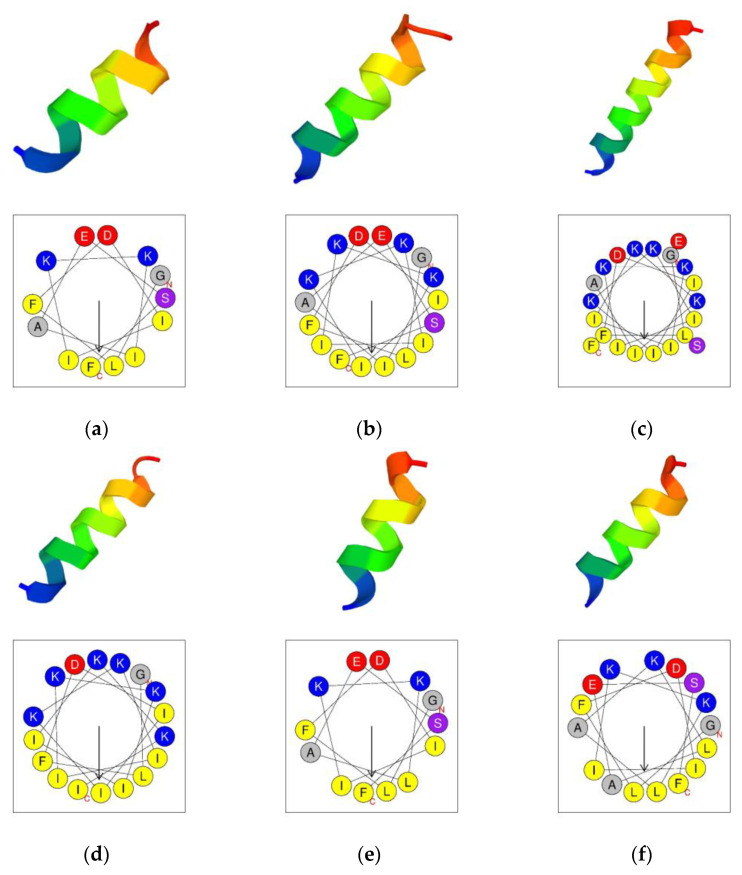
The predicted 3D secondary structure of the peptides from PEP-FOLD3 (above) and the helical wheel projection of the peptides (under). (**a**) aurein 1.2; (**b**) IK-1; (**c**) IK-2; (**d**) IK-3; (**e**) KLA-1; and (**f**) KLA-2. The helices are rainbow-colored with N-terminus (blue) and C-terminus (red). In the helical wheels, the C-terminals and N-terminals are indicated by “C” and “N”, respectively. The arrow in the middle of the circle indicates the hydrophobic direction formed by amino acids with high hydrophobicity. The yellow circles represent the hydrophobic amino acids. The grey circles represent the neutral amino acids. The blue circles represent the polar amino acid residues.

The secondary structures were predicted by using the PEP-FOLD3 program [27]. The results indicated that aurein 1.2 and its analogs all tended to form an α-helical structure (Figure 2). The helical wheels were acquired by using the Heliquest program (Figure 2) [28]. The helical wheels indicated that all the peptides had a considerable hydrophobic face which contained a long cluster of hydrophobic residues.

**Figure 3:**
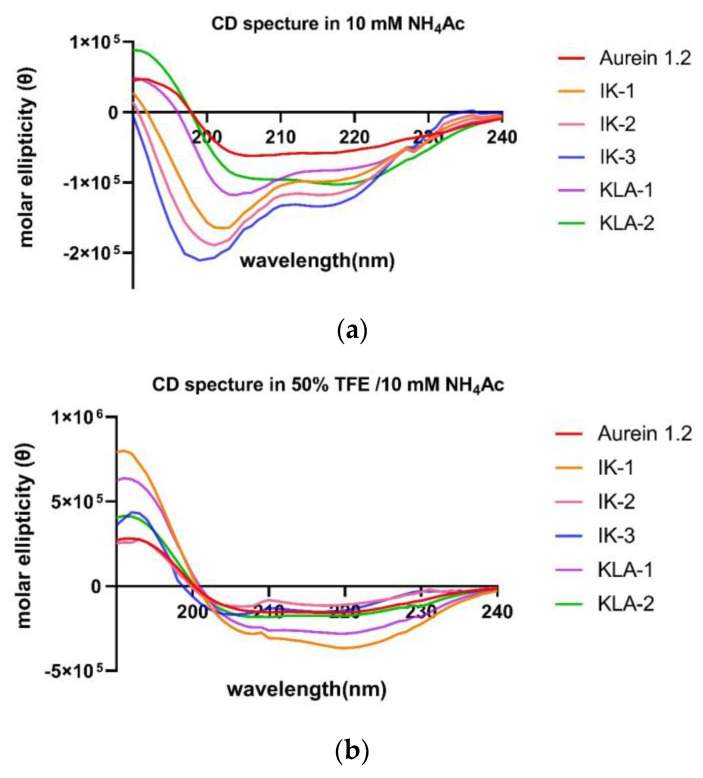
CD spectra of aurein 1.2 (red); IK-1 (orange); IK-2 (pink); IK-3 (blue); KLA-1 (purple) and KLA-2 (green) in (**a**) 10 mM NH_4_Ac solution and (**b**) 50% TFE/10 mM NH_4_Ac solution.

The secondary structure confirmation was performed in a circular dichroism study; 10 µM NH_4_Ac mimicked the aqueous environment. In this, all the peptides tended to form α-helical structures, and in a 50% tetrafluoroethylene (TFE) environment, which mimics the membrane environment, all peptides still demonstrated α-helical structures (Figure 3).

### 2.3. MIC and MBC Assays

The antimicrobial activity screening was carried out by using three types of Gram-positive bacteria and three types of Gram-negative bacteria. The results indicated that the parent peptide, aurein 1.2, possessed moderate antimicrobial activity against Gram-positive bacteria (MICs from 8 to 32 μM). For Gram-negative bacteria, the antimicrobial abilities were marginally weaker than those against Gram-positive bacteria. For the modified analogs in general, all exhibited activity improvement against Gram-negative bacteria. For IK-1, a significant improvement in inhibition of *E. faecalis* was demonstrated, and this analog was the most outstanding among all the tested peptides, possessing 4-fold more potency than aurein 1.2. The antimicrobial activities against *E. coli* and *P. aeruginosa* were substantially stronger than those of the parent peptide. Even so, IK-1 was almost non-functional against *K. pneumoniae*. The antimicrobial activity of IK-2 did not expectedly increase against Gram-positive bacteria. Compared to the parent peptide, aurein 1.2, it presented a slight increase in activity against *E. coli* and *K. pneumoniae*. For IK-3, the antimicrobial activity against Gram-positive bacteria was essentially the same as that of the parent peptide. The anti-Gram-negative bacterial function was 4–8-fold greater than that of the parent peptide.

The antibacterial activity of KLA-1 changed 1–2-fold, showing a slight enhancement against *E. coli* and *P. aeruginosa*. KLA-2 demonstrated the greatest enhancement of antimicrobial activity among all the tested peptides. For Gram-positive bacteria, the MIC value was improved 2–4-fold, reaching 2 μM for *S. aureus*, and this was the only peptide that showed a significant improvement in action against Gram-positive bacteria. When applied to Gram-negative bacteria, its antimicrobial activity showed a 4–16-fold improvement compared to the parent peptide. All the MIC and MBC values are shown in Table 2.

### 2.4. Prevention and Eradication of Bacterial Biofilm

The effect of the peptides on preventing biofilm formation and eradication of existing biofilm demanded a relatively high concentration. Firstly, the parent peptide, aurein 1.2, demonstrated a moderate ability in the prevention of biofilm formation and required a high concentration to be effective on Gram-negative bacteria (64–128 μM). When applied to Gram-positive bacteria, especially for *S. aureus* and MRSA, it had a relatively higher potency with an MBIC value of between 16 to 32 μM. 

For IK-1, generally, the MBIC and MBIC values were basically the same as for aurein 1.2., although a doubling of potency was observed for the prevention of *E. faecalis* and *P. aeruginosa* biofilms. As for IK-2, it had no effects on either biofilm prevention or eradication. Overall, the ability of IK-3 to clear existing biofilm showed little improvement compared to the parent peptide. However, its effect on *E. coli* biofilm was notable, both in terms of prevention and removal, as it produced a two-fold enhancement over the parent peptide. For KLA-1, it has similar effects on both the MBIC and MBEC values as compared to aurein 1.2. KLA-2 was the most active peptide in the prevention of biofilm formation and achieved a 2–8-fold enhancement over its MBIC value. The efficacy of this peptide against Gram-positive bacteria also increased with MBIC values ranging from 2 to 16 μM. All MBIC and MBEC values are listed in Table 3.

### 2.5. Killing Kinetics Assessment

The killing kinetics assessment was performed using *S. aureus* and *E. coli* with the respective most potent peptides against each bacterium.

When applied to *S. aureus*, all peptides did not show a completely bactericidal effect after 120 min. However, according to the MBC values, all the tested peptides had the bactericidal ability at their MIC values. This meant that the time these peptides needed for full bactericidal activity was longer than 120 min, and this was a very slow bactericidal rate. IK-1 and KLA-2, however, both demonstrated significant inhibition of bacterial growth.

Aurein 1.2, at 2-fold its MIC value, was not fully bactericidal at 120 min. Nevertheless, IK-1 and KLA-2 exhibited a dramatic increase in their bactericidal rates. As for IK-1, it displayed a complete kill after 30 min while KLA-2 achieved a 100% bactericidal effect after 10 min. 

**Figure 4:**
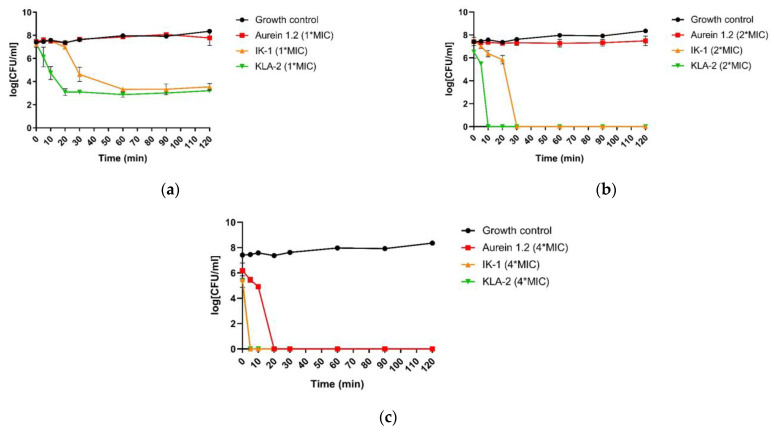
The kinetic killing curves of aurein 1.2, IK-1, and KLA-2 against *S. aureus* (ATCC 6538) at (**a**) MIC, (**b**) 2-fold MIC, and (**c**) 4-fold MIC. The error bars in the graphs around mean data points indicated the standard error of the mean (SEM) for each set of data in the nine replicates from three experiments.

At a 4-fold MIC concentration, aurein 1.2 exhibited a rapid killing in 20 min, and it was observed that the overall killing rate of aurein 1.2 was not high, and this showed a close correlation with the concentration. When the concentration was increased, the killing rates of IK-1 and KLA-2 showed some improvement, completing the kill in 5 min. The killing kinetic curves of the peptides against *S. aureus* are shown in Figure 4.

**Figure 5:**
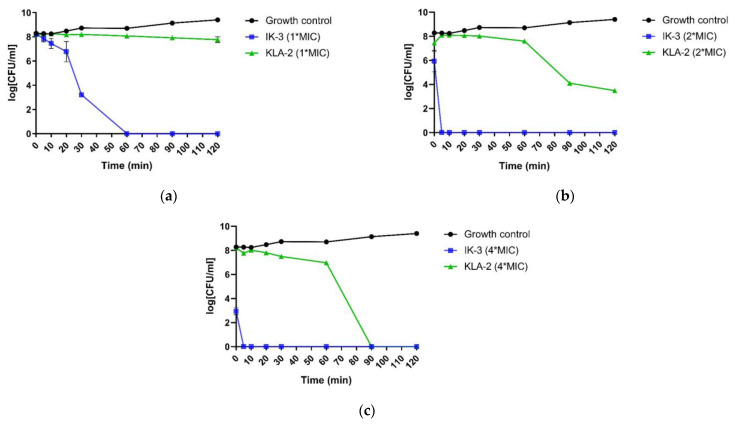
The kinetic killing curves of IK-3 and KLA-2 against *E. coli* (NCTC 8739) at (**a**) MIC, (**b**) 2-fold MIC, and (**c**) 4-fold MIC. The error bars in the graphs around mean data points indicate the standard error of the mean (SEM) for each set of data in the nine replicates from three experiments.

For application to *E. coli*, the most potent peptides, IK-3 and KLA-2, were chosen in this study. The results indicated that IK-3 presented a complete killing to *E. coli* at its MIC concentration in 60 min, which correlated to its MBC value. When the concentration was increased, the killing rate was dramatically improved. At 2-fold MIC, the killing was completed in 5 min. At 4-fold the MIC value, the bacteria were effectively inhibited at the moment the peptide was applied, finishing the bactericidal effect after 5 min. The scenario was totally different with KLA-2. Similar to its effect on *S. aureus*, when applied to *E. coli*, the bactericidal activity was sluggish. This peptide did not demonstrate a complete killing at MIC and 2-fold MIC after 120 min, although a significant inhibition effect was observed at 2-fold MIC concentration. At 4-fold MIC, its killing rate was still slow, accomplishing complete effect after 90 min. The kinetic killing curves of tested peptides against *E. coli* are shown in Figure 5. 

### 2.6. SYTOX Green Permeability Assay

SYTOX green is an exogenous fluorescent nucleic acid stain that can be utilized to measure the integrity of bacterial membranes. The combination of SYTOX green and nucleic acid results in intense fluorescence emission and thus reveals the loss of bacterial cell membranes [29]. Melittin is a 26-residue membrane-active peptide with a bent rod or helix-hinge-helix secondary structure [30]. Although the mechanism of action is still controversial, the hydrophobic and amphiphilic properties allow melittin insertion into the bilayer to significantly reduce the permeability barrier by separating the polar and non-polar portions of the bilayer [31]. Melittin was selected as a positive control for this assay due to its strong membrane-breaking ability.

Aurein 1.2, IK-1, and KLA-2 were selected in the SYTOX green membrane permeability study of *S. aureus*. After 120 min, all peptides did not affect 100% membrane permeability at their respective MICs; for aurein 1.2 and KLA-2, low membrane permeability rates of around 50% and 40% were observed, respectively. The membrane permeability of IK-1 showed a surge before 30 min, which correlated with the time-killing curve, but no peptide caused complete permeability of *S. aureus* cell membranes. 

**Figure 6:**
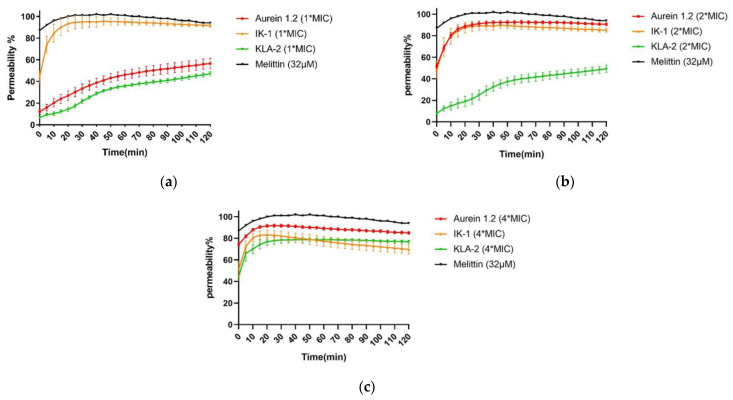
Permeability curves of aurein 1.2, IK-1, KLA-2, and melittin against *S. aureus* (ATCC 6538) at (**a**) MIC, (**b**) 2-fold MIC, and (**c**) 4-fold MIC. The total percentage (100%) of membrane permeabilization was achieved by use of melittin (32 μM).

When the concentration was raised 2-fold, aurein 1.2 showed a dramatic increase in its permeability, reaching around 90% within 20 min. IK-1 and KLA-2 showed the same tendencies. However, the permeability of KLA-2 did not increase significantly until a 4-fold MIC, at which around 70% occurred within 120 min. The permeability rate of IK-1 was not closely related to its concentration and did not change significantly at different concentrations. All the membrane permeability curves are shown in Figure 6.

IK-3 and KLA-2 were used for the *E. coli* membrane permeability study. At their MICs, almost no membrane permeabilization was detected. With reference to the killing-time curves of these peptides, the situations were totally different. IK-3 completed bacterial killing in 60 min, while KLA-2 did not produce a significant bacterial kill within 120 min, which means the action mechanism of IK-3 might not be based on the membrane disruption at lower concentrations. 

At 2-fold MICs, the membrane lysis rates of IK-3 and KLA-2 slightly increased, reaching 50% and 40%, respectively. Compared with the killing-time kinetic curves, for IK-3, even though the membrane lysis rate was only around 50%, the bactericidal action was completed in 5 min, further confirming that there may be more than one mechanism of IK-3 action on *E. coli*. KLA-2 showed a 40% permeabilizing effect at 2-fold MIC within 20 min, which could be correlated with the killing-time curve. Membrane permeabilizing ability may thus be an important mechanism of action for KLA-2. 

**Figure 7:**
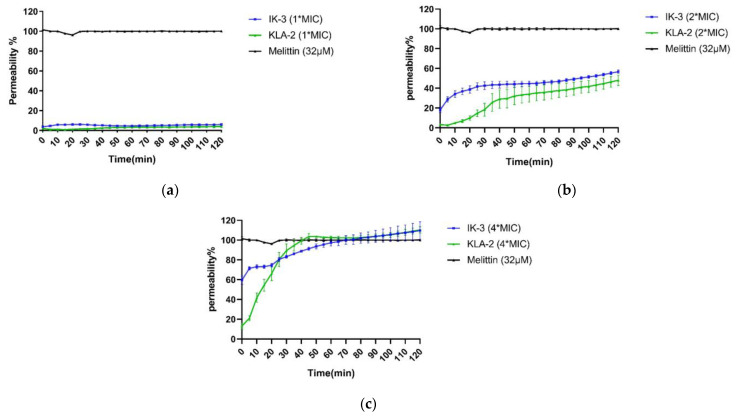
Permeability curves of IK-3, KLA-2, and melittin against *E. coli* (NCTC 8739) at (**a**) MIC, (**b**) 2-fold MIC, and (**c**) 4-fold MIC. The 100% membrane permeabilization was measured using bacteria treated with melittin (32 μM).

At 4-fold MIC, the lytic rate of IK-3 dramatically increased from 60% instantly to 100% in 120 min. KLA-2 also had a high lytic percentage on the cell membrane and finally caused complete permeabilization. All membrane permeability curves are shown in Figure 7. 

### 2.7. MTT Anti-Proliferation Activity

In the antiproliferation study, three cancer cell lines were employed. For the lung cancer cell line, H838, the parent peptide aurein 1.2 exhibited a moderate antiproliferation activity with an IC_50_ value of 26.94 μM. After an -IIKK- region was added to IK-1, the antiproliferation activity increased significantly. The IC_50_ value was reduced by around 7.7-fold. However, the introduction of a second -IIKK- region into IK-2 did not have the dramatic positive effect expected in that its antiproliferation activity was only slightly affected. After moving the -AESF- region on IK-3, its activity achieved its best value among all the tested peptides. The IC_50_ value of IK-3 was 2.99 µM, reduced 9-fold compared to its parent peptide, aurein 1.2. For KLA-1, it displayed a weaker activity after modification. Even the replacement of isoleucine with leucine in KLA-1 did not have a positive influence; the addition of another -KLA- region, based on KLA-1, also improved the anticancer activity of KLA-2, attaining a 5.9-fold better efficacy. 

Generally, the activity of peptides on the MCF-7 cell line was marginally better than that observed with the H838 cell line. Similar anticancer activity was also found against the human breast cancer cell line MCF-7. The difference was that the potency of IK-3 did not increase further after modification. Moreover, KLA-1 demonstrated better activity after the substitution with an IC_50_ 1.47-fold lower than the parent peptide. Based on this, KLA-2 also improved with an IC_50_ of 3.55 μM.

**Figure 8:**
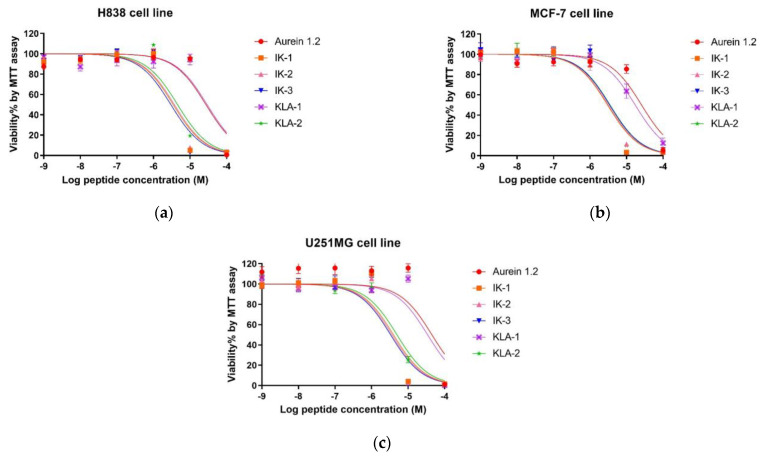
The effects of aurein 1.2 and its analogs on the proliferation of human cancer cell lines. (**a**) H838, non-small-cell lung cancer; (**b**) MCF-7, human breast cancer cell line; (**c**) U251MG, human glioblastoma astrocytoma. The cancer cells were treated with a series of peptide concentrations from 10^−9^ M to 10^−4^ M; 0.1% Triton X-100 was used as the positive control. The curves were fitted using normalized dose–response analysis. The error bar represents the means ± standard deviation of three independent experiments.

As for the human glioblastoma astrocytoma cell line, U251MG, the inhibition of this cell line seemed to be the most difficult. Aurein 1.2 demonstrated the highest IC_50_ value of 38.41μM among all the tested cell lines. The effect of all peptides had a similar tendency to that observed for H838 cells. IK-3 had the most potent effect on the U251 cell line. All dose-response curves are shown in Figure 8, and the calculated IC_50_ values are shown in Table 4.

### 2.8. Haemolytic Activity Evaluation

The hemolytic activity of parent peptide aurein 1.2 was relatively high, with an HC_50_ value at around 30 μM. The hemolytic activities of the peptides, at their MIC values for *E. coli*, are listed in Table 5 to further illustrate the peptide selectivity. At the MIC value for *E. coli*, aurein 1.2 showed a 20.56% hemolytic activity against horse erythrocytes. However, the introduction of the membrane-penetrating -IIKK- further reduced the peptide selectivity. IK-1 and IK-2 possessed the strongest hemolytic activity among all the peptides, exhibiting 58.11% and 53.56%, respectively, at their MIC values. The movement of the -AESF- domain reduced the hemolytic activity of the peptide to a certain extent. The activity at its MIC value decreased significantly to 15.51%.

**Figure 9:**
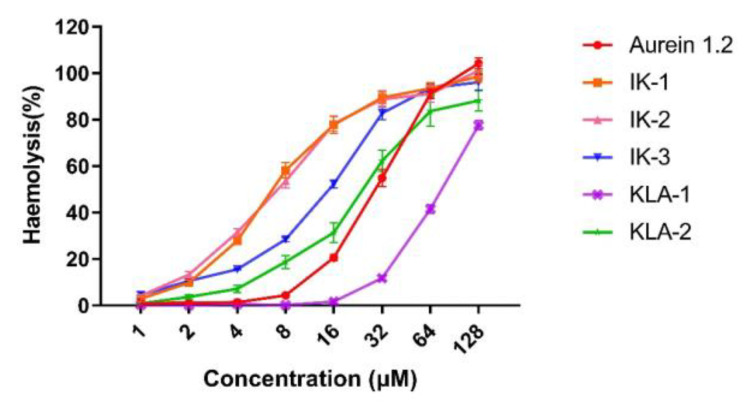
Hemolytic activity of aurein 1.2 and its analogs against horse blood cells. The percentage of hemolysis was calculated using 1% TritonX-100 as the positive control. Phosphate-buffered saline (PBS) was applied as the negative control. The error bars represent the SEM (standard error of the mean) (*n* = 9).

The substitution of isoleucine with a leucine residue gave the peptide a higher selectivity. The hemolytic activity of KIL-1 was the weakest among all the peptides. At MIC values, almost no hemolysis was detected. For KLA-2, hemolytic activity also decreased greatly compared to parent peptide aurein 1.2, to around 3.67% at its MIC value. The hemolysis curves are shown in Figure 9. 

## 3. Discussion

HDPs, as promising antimicrobial agents which are unlikely to induce drug resistance, are regarded as effective weapons in bacterial-related infections and cancer therapy [32]. However, some undesired properties, such as low in vivo efficacy and potential toxicity, have complicated the development of the peptides for clinical use [12]. De novo and rational design of new peptides with better characteristics has become possible because of advancements in peptide synthesis and high-throughput activity screening [33]. In this study, aurein 1.2 was chosen as a template peptide. Aurein 1.2 was first isolated from the skin secretion of the southern bell frogs, *Litoria aurea* and *Litoria raniformis,* by Rozek [15]. Some research on this peptide has demonstrated that aurein 1.2 has broad-spectrum antimicrobial and anticancer properties, though these activities were moderate [15,34]. Thus, the rational design of analogs appeared to be a relevant approach to improve properties for the future development of aurein 1.2 as an efficacious drug template. 

In this study, five aurein 1.2 analogs were designed, aiming to achieve more potent bio-activities. In the antimicrobial activity evaluation, among all the IK analogs, IK-3 demonstrated the most potent ability for the growth inhibition and bactericidal activity of Gram-negative bacteria. On the contrary, by further modification, the MIC value of IK analogs against Gram-positive bacteria did not change or indeed decrease compared to the parent peptide. The inverse tendencies of the analogs’ effects on different types of bacteria were obvious. The chemical structural differences in surfaces between Gram-positive bacteria and Gram-negative bacteria are key factors leading to different mechanisms. 

The cell wall is a structure that exists in both Gram-positive bacteria and Gram-negative bacteria, although the thickness of the cell wall in different types of bacteria is varied. Unlike Gram-negative bacteria, which have a single layer of the cell wall that is just 7–8 nm thick, Gram-positive bacteria have numerous peptidoglycan (PGN) layers that are roughly 40–80 nm thick [35]. Teichoic acid polymers, which significantly contribute to a negative bacterial surface charge, are incorporated into the cell envelopes of the majority of Gram-positive bacteria. Teichoic acid’s fundamental structure consists of a soluble polymer of glycerolphosphate or ribitolphosphate repeating units that are either covalently bonded to PGN (wall teichoic acid, WTA)’s *N*-acetylmuramic acid or tethered to the cytoplasmic membrane through a glycolipid anchor (lipoteichoic acid, LTA) [36]. Every repeat unit contains a negative charge from the phosphate group, which can attract the positively charged antimicrobial peptides. However, the interaction with the negatively charged LTA might decrease the effective concentration in the cytoplasm membrane, which plays an essential role in the destabilization of the membrane and bacterial killing. In our case, all the IK analogs presented an increased net charge, which might increase the affinity of peptides to the LTA, decreasing the peptide concentration on the plasma membrane, which is necessary for killing, thereby decreasing the activity of Gram-positive bacteria. 

Among all the peptides, KLA-2 demonstrated the most potent antimicrobial activity. Reviewing the physiochemical parameters, KLA-2 had the lowest hydrophobic moment as well as a relatively low hydrophobicity among all the tested peptides. The hydrophobic moment is closely related to the peptides’ α-helical structure formation and the insertion of the peptides into the bacterial cell membrane. The study of peptide hydrophobic moment compared to its bioactivity, carried out by Takechi, showed clearly the effect of this parameter [37]. Normally, the higher hydrophobicity and amphipathicity are regarded as stabilization factors, prolonging the lifetime of the pores that AMPs form. However, in this study, the analogs with lower hydrophobic moments demonstrated a longer duration and charge flux from stable membrane pores. Therefore, the increased amphipathy might not always lead to an increased penetrating effect but to the cytotoxicity related to destabilized integrity of the cell membrane. Therefore, the optimal way for the peptides with low hydrophobic moment to penetrate cells is by plasma membrane perturbation instead of membrane pore stabilization. In our study, KLA-2 showed potent bio-activity and selectivity. However, the membrane permeability data showed that KLA-2 did not have a strong effect on *S. aureus* and *E. coli* cell membranes at low concentrations. The permeability percentages were only 40% and 3%, respectively, at the MIC, which is consistent with the conclusion of Takechi’s study. Penetration while maintaining membrane integrity should be considered the killing mode of KLA-2. 

The bactericidal rate is also an important parameter that reflects the clinical potential of the peptides. For the killing of *S. aureus*, the killing kinetic curves illustrated that the parent peptide demonstrated a low killing rate. After the introduction of different cell-penetrating sequences, the killing rate of the peptides increased significantly. In Zhang’s study, a structure–activity relationship study was carried out to determine the effective parameters of the bactericidal rate of peptides [38]. With the increase in the hydrophobicity, the bacterial binding rate, LPS neutralization, and permeabilization of the inner and outer membrane will also increase. In this study, aurein 1.2, IK-1, and KLA-2 contained 53.85%, 52.94%, and 56.25% of hydrophobic residues in their sequences, respectively. KLA-2, which has the highest hydrophobic residue ratio and the longest cluster of hydrophobic faces, demonstrated a more rapid killing rate compared to the other peptides. 

In the anti-proliferation study, the IIKK-modified peptides displayed an overall better activity than the KLA-modified analogs. The -IIKK- region, which was studied in the designed peptide G(IIKK)3I-NH_2_, is widely used in anticancer peptide modification as a cell-penetrating region. The selective reactions of the peptides to various cell membranes through the balance between electrostatic contact and hydrophobic effect have been proven to be the source of the common discriminative responses to bacterial and cancer cells. G(IIKK)_3_I-NH_2_ demonstrated a rapid binding driven by its anionic characteristics and the highly unsaturated lipid chains, ensuring a high permeability rate to the cancer cell. In addition, a study on the number of repeats in the -IIKK- region of G(IIKK)_n_I-NH_2_ (*n* = 1–4) also showed that, when the -IIKK- region was repeated three times, the peptide showed the most potent bio-activity against cancer cells and an ideal selectivity [39]. Moreover, a terminal amino acid modification study of (IIKK)_3_-NH_2_ has been carried out by Chen [17]. This study aimed to study the N-terminal and C-terminal amino acids and their effect on peptide functions. Four analogs were investigated. Among all the tested peptides, ((IIKK)_3_-NH_2_, G(IIKK)_3_-NH_2_, (IIKK)_3_I-NH_2_, G(IIKK)_3_I-NH_2_), the analog with isoleucine at the C-terminus without glycine at the N-terminus ((IIKK)_3_I-NH_2_), possessed the most potent function without cytotoxicity. Therefore, in our study, as aurein 1.2 already has a single -IIKK- region in its sequence, either one or two IIKK regions were introduced. After the insertion of the -IIKK- region, the modified peptide did show an approximately 11-fold stronger anticancer activity compared to the parent peptide. However, the triple repeat analog, IK-2, did not exhibit a better function compared to IK-1. The possible reason for this is that IK-1 possessed a significantly more helical structure than IK-2. For IK-3, the removal of the C-terminal amino acid, making a glycine at the C-terminus, did provide IK-3 with the strongest anti-proliferation activity, which corresponds to a previous study. 

The strength of a peptide’s hemolytic action is related to many factors, including length, sequence, charge, helicity, hydrophobicity, amphipathicity, the hydrophobic/hydrophilic angle, and self-association. These parameters are not independent, and they will impact each other and together determine the hemolytic potential of a peptide [40]. Benincasa identified the relationship between peptide length to hemolysis [41]. The result indicated that the melittin fragment peptide with 18 amino acids demonstrated 5–7-fold less potency in its antimicrobial function but 300-fold less hemolytic activity compared to melittin. In this study, KLA-1 presented the lowest hemolytic activity. The possible reason for this is that KLA-1 had the shortest chain length and net charge, producing a higher selectivity. 

Overall, in this study, the cell penetration regions -IIKK- and -KLA- were selected as examples to focus on lysine-based modifications, providing analogs of broad-spectrum multifunctionality and producing potential leads for subsequent clinical evaluation and perhaps application. However, membrane penetration regions are diverse, and a large number of arginine- and tryptophan-based membrane penetration regions are now also widely studied [42,43]. In this study, other amino acid-based membrane penetration regions were not mentioned. 

## 4. Materials and Methods

### 4.1. Peptide Synthesis, Purification, and Identification 

Aurein 1.2 and its designed analogs were synthesized by soli-phase peptide synthesis (SPPS) in a Tribute^TM^ automated solid-phase peptide synthesizer (Protein Technologies, Tucson, AZ, USA). To synthesize 0.3 mmol of peptide, 0.75 mmol of amino acid was required. All the amino acids were weighed accurately, together with the activator, HBTU, and Rink amide MBHA resin, and were loaded into the machine and prior to peptide synthesis. After the synthesis procedure was completed, the peptides were cleaved by cleavage solution which contained 94% Trifluoroacetic acid (TFA), 2% thioanisole (TIS), 2% 1,2-ethanedithiol (EDT), and 2% H_2_O, by stirring 2 h. Afterward, the washing procedure was conducted three times by using 30 mL of diethyl ether each time. Then, the peptides were dissolved in 15 mL of buffer A (0.05% TFA and 99.95% water) and 15 mL of buffer B (0.05% TFA, 19.95% water, and 80% acetonitrile) before they were lyophilized.

Crude peptides were purified by reverse-phase HPLC (LUNA C-5 preparation column, 250 × 10 mm, Phenomenex, UK) with a linear gradient from 100% buffer A (0.05/99.5 (*v/v*) TFA/water) and 0% buffer B (0.05/19.95/80.00 (*v/v/v*) TFA/water/acetonitrile) to 0% buffer A and 100% buffer B in 80 min, at a flow rate of 1mL/min. The masses of the peptides were verified by HPLC (Supplementary in Figure S1) and MALDI-TOF MS (Voyager DE, perceptive Biosystems, Framingham, MA, USA), using CHCA (10 mg/mL; in 70% acetonitrile, 0.02% TFA and 30% water) as matrix in positive detection mode.

### 4.2. Physicochemical Property Analysis, Secondary Structure Prediction, and Confirmation

Physicochemical properties of aurein 1.2 and its analogs were acquired via the Heliquest program (https://heliquest.ipmc.cnrs.fr/cgi-bin/computparams.py, accessed on 15 August 2022). Secondary structure prediction was carried out using the PEP-FOLD3 program (https://bioserv.rpbs.univ-paris-diderot.fr/services/PEP-FOLD3/, accessed on 11 August 2022). The helical wheel projections were created by the Heliquest program (https://heliquest.ipmc.cnrs.fr/cgi-bin/computparams.py, accessed on 16 August 2022).

The secondary structure was determined by circular dichroism using a JASCO J-815 CD spectrometer (Jasco, Essex, UK). All the peptides were prepared at a concentration of 50 μM in 10 mM NH_4_Ac and 50% TFE (*v/v*, in 10 mM NH_4_Ac). Samples were loaded into a 1mm quartz cuvette and then analyzed at 20 °C, in a scan range of 190–250 nm, scan speed of 100 nm/min, 1nm bandwidth, and 0.5 nm data patch. The baseline-subtracted raw data were converted to mean residue molar ellipticity and further analyzed by the online tool, BeStSel (https://bestsel.elte.hu/index.php, accessed on 14 November 2022). 

### 4.3. Mean Inhibitory Concentration (MIC) and Mean Bactericidal Concentration (MBC) Assays

The antimicrobial activity evaluation was conducted by use of MIC and MBC assays, using three Gram-positive bacteria and three Gram-negative bacteria. The peptides were dissolved in dimethyl sulfoxide (DMSO) for a final concentration series from 1 to 128 µM; 20 µmol of norfloxacin was applied as the positive control, and one µL of DMSO was used as the negative control. The bacteria were inoculated in the corresponding cultural media overnight and subcultured to the desired OD value (OD = 0.23 for Gram-positive bacteria, OD = 0.4 for Gram-negative bacteria corresponding to 5 × 10^5^ CFU/mL); 20 mL of culture broth and 100 µL of subcultured bacteria were mixed before being loaded onto the plate, and 1 µL of the peptide cocktail was mixed with 99 µL of diluted bacteria in 96 wells plates and then was incubated for 20 to 24 h at 37 °C. Afterward, the OD value (λ = 550 nm) of each well was detected using a Synergy HT plate reader (Biotech, Minneapolis, MN, USA). The MIC value was the lowest concentration without bacterial growth visually. Subsequently, 10 µL of the solution in each clear well was loaded onto an agar plate and cultured for 20 h. The lowest concentration without colony growth was regarded as the MBC.

### 4.4. Mean Biofilm Inhibition and Mean Biofilm Eradication Concentration (MBIC/MBEC) Assays

The minimum biofilm inhibition and minimum biofilm eradication concentrations were obtained by use of the MBIC and MBEC assays. The anti-biofilm activity of the peptides was evaluated by use of *S. aureus* (ATCC 6538), MRSA (NCTC 12493), *E. faecalis* (NCTC 12493), *E. coli* (ATCC CRM 8739), *K. pneumoniae* (ATCC43816), and *P. aeruginosa* (ATCC CRM 9027).

For the MBIC assay, the bacteria were cultured in tryptic soy broth (TSB) broth with 1% glucose for Gram-positive bacteria biofilm and Luria Bertani (LB) broth with 1% glucose for Gram-negative bacteria biofilm. Bacteria were inoculated, subcultured, and diluted in the same method described before. The peptides were prepared in a concentration of 1–128 µM; 1 µL of peptide and 99 µL of subcultured bacteria were mixed in 96 well plates and cultured at 37 °C with a shear force of 200 rpm in a humid environment for 20–24 h. Then, the plate was washed with 130 µL of PBS twice, and 130 µL of methanol was added to stabilize the biofilm overnight; then, the plate was dyed with 100 mL of 0.5% crystal violet for 15 min. The stained biofilm was then washed once more and allowed to dry overnight. The stain was dissolved in acetic acid at a 30% concentration, and it was shaken for 15 min to ensure homogeneity. A Synergy HT plate reader was then used to read the plate at 595 nm. The MBIC value was defined as the lowest concentration at which biofilms were not formed.

In the minimum biofilm eradication essay, the biofilm was first formed in the 96 well plates by loading 100 µL of the bacteria to the plate wells and culturing at 37 °C for 20–24 h. Then, the plate was washed with 130 µL of PBS twice. The peptides were prepared in the same concentration series, and then 1 µL of the peptide was mixed with 99 µL of the corresponding broth medium before it was loaded onto the plate. The plate was cultured under the same conditions as described before. After 20–24 h of culture, the biofilm was rinsed, stabilized, and stained. The minimum concentration of peptide at which biofilm was completely eradicated was defined as the MBEC of the peptide. 

### 4.5. Killing-Time Kinetics Assessment

The killing-time experiment was used to determine the peptides’ kinetics of killing. *S. aureus* and *E. coli* were chosen and cultivated with various peptide concentrations (MIC, 2-fold MIC, and 4-fold MIC). The bacteria were inoculated, subcultured, and diluted as in the method described before. Ten µL of bacteria at the selected time points (0, 5, 10, 20, 30, 60, 90, and 120 min) were mixed with 90 µL PBS to achieve 10-fold dilutions. The dilution step was repeated three times until the concentration was 10^−4^. Then, 10 µL of the mixed cocktail was placed onto an agar plate to count and record the number of living bacteria after incubating at culture temperature for 20–24 h. The CFU/mL could be calculated using the formula CFU/mL = (number of colonies × dilution factor)/volume of culture.

### 4.6. SYTOX Green Permeability Study

The SYTOX green permeability assay was used to determine the peptides’ disruptive impact on the bacterial cell membrane by use of the SYTOX Green Nucleic Acid Stain (Thermo Fisher Scientific, Waltham, MA, USA). The bacteria were cultured in their corresponding culture media overnight and then subcultured by adding 500 µL of bacteria to 25 mL of the medium at 37 °C degrees for 1.5 h in a shaking incubator. Subsequently, the bacteria were collected at 4 °C by centrifugation at 1000× *g* and washed with 30 mL of 5% TSB (in 0.85% NaCl) solution twice. The collected bacteria were resuspended using 5% TSB solution to obtain an OD value of 0.68–0.72 for *S. aureus* and an OD value of 0.65–0.7 for *E. coli* at 590 nm. Then, 50 μL of bacteria and 40 μL of peptide (concentration = MIC, 2 × MIC, and 4 × MIC), in conjunction with 10 μL of SYTOX green (50 μM in 5% TSB, Life Technologies, Cheshire, UK), were loaded into the wells of a black 96 well plate then placed in a Synergy HT plate reader set to excitation and emission wavelengths of 485 nm and 528 nm, respectively at 37 °C for 120 min (interval 5 min). The percentage of cell permeabilization could be calculated by comparing the fluorescent density of the bacterial suspension treated with 32 μM of the membrane-disrupting peptide, melittin, as positive control and 5% TSB in 0.85% NaCl as growth control. The rate of permeability was determined with the following equation: Permeability (%) = (A or A0 − Aa)/(Ap − Aa) × 100%. “A” is the average OD value of the sample group; “A0” is the average OD value of the negative control group; “Ap” is the average OD of the positive control group; “Aa” is the average OD of the additional control group.

### 4.7. Anti-Proliferation Study

The MTT test measures the anti-proliferation of the peptide by measuring MTT conversion by live cells into a formazan that cannot be dissolved in water. In this section, three cancer cell lines: human non-small-cell lung cancer cell line, NCI-H838 (ATCC-CRL-5844); human glioblastoma astrocytoma cell line, U251MG (ECACC-09063001) and human breast cancer cell line, MCF-7 (ATCC-HTB-22), were selected for the anti-proliferation study. H838 cells were cultured in Roswell Park Memorial Institute (RPMI)-1640 medium (Life Technologies, Paisley, UK) mixed with 10% (*v/v*) fetal bovine serum (FBS) and 1% penicillin-streptomycin (Gibco, 10,000 U/mL). U251MG and MCF-7 cells were cultured in Dulbecco’s Modified Eagle medium (DMEM) (Life Technologies, Carlsbad, CA) mixed with 10% (*v/v*) FBS and 1% penicillin–streptomycin (Gibco, 10,000 U/mL). The cancer cells were first seeded into the 96-well plates at a cell density of 8000 per well for 20–24 h at 37 °C. Then, the cells were starved by switching the FBS containing complete growth medium to FBS lacking serum-free medium for 4 h. Peptides were dissolved to a series of final concentrations from 10^−9^ to 10^−4^ M in ddH_2_O. After starvation, 1 μL of the peptide with 99 μL of the serum-free medium was added to the plate and then cultured for 20–24 h.; 10 µL of MTT (3-(4,5-dimethylthiazol-2-yl)-2,5-diphenyltetrazolium bromide, 5 mg/mL, Sigma, UK) were loaded into each well in the dark and incubated at 37 °C. Subsequently, the liquid in each well was removed completely before 100 μM of DMSO was added to each well to dissolve the formazan. The plate was read at 570 nm using a Synergy HT plate reader.

### 4.8. Haemolytic Activity Evaluation

The hemolytic activity of the peptides on mammalian erythrocytes was examined using 2% defibrinated horse blood (TCS Biosciences Ltd., Buckinghamshire, UK). Firstly, the defibrinated horse blood was washed; 2 mL of horse blood was added into a 50 mL tube; then, 30 mL of sterilized PBS was added dropwise into the tube, and the tube was mixed in a shaking bed and then centrifuged at 930× *g* for 10 min. The procedure was repeated until the supernatant of the centrifuge tube was clear. Afterward, the blood cell pellet was dispersed to make a 4% suspension (*v/v*) by adding 50 mL of sterilized PBS. Peptides were dissolved using PBS to achieve final concentrations of 1–128 μM and then mixed with the blood cell suspensions in 1.5-mL tubes. The blood cell suspensions treated with 0.1% Triton X-100 and PBS were used as positive controls and negative controls, respectively. Then the peptide-blood mixture was incubated at 37 °C degrees for 2 h in an incubator. After incubation, the tubes were centrifuged at 930× *g* for 10 min, and 100 µL of the supernatants were loaded into wells of the 96-well plates. The absorbance was obtained by Synergy HT plate reader at 490 nm. The hemolytic activity was calculated using the formula, hemolysis = (absorbance of experimental groups − absorbance of negative control))/(absorbance of positive control − absorbance of negative control) × 100%.

### 4.9. Statistical Analysis

Statistical analysis of all bioactivity assay data was performed by use of Prism (GraphPad Software, San Diego, CA, USA). One-way ANOVA was used to analyze the statistical significance of the differences. The data points represent the mean of the independent experiments; the error bars represent the standard error (SEM) of the mean, ns means non-significance difference, * means *p* < 0.5, ** means 0.001 < *p* < 0.01, *** means *p* < 0.001, and **** means *p* < 0.0001.

## 5. Conclusions

In conclusion, in this study, a broad-spectrum multi-biofunctional peptide, aurein 1.2, was chosen as a template for the design and synthesis of analogs with superior activities. Cell-membrane-penetrating peptide motifs, -IIKK- and -KLA-, were introduced into the natural template sequence to hopefully increase the peptide’s functionality and selectivity. The analog, KLA-2, demonstrated an outstanding broad-spectrum antimicrobial activity as well as an anti-biofilm efficacy and represented an interesting lead for a potential antimicrobial agent. With respect to cell anti-proliferation effects, this analog also demonstrated a potent functionality. However, the killing of Gram-negative bacteria was slow, and hemolytic effects were relatively high, which are aspects in need of attention through future structure/activity studies. Moreover, the cytotoxicity study in this project was not thorough, and the toxicity of analogs to normal cell lines was not included. Further research thus needs to be carried out in the future.

## Figures and Tables

**Table 1:** Peptide sequences and their physiochemical parameters. The bold and underlined letters indicate the inserted amino acids in the parent peptide sequence.

Peptide Name	Peptide Sequence	Theoretical Molecular Weight/g/mol	Hydrophobicity/H	Hydrophobic Moment/µH	Charge
aurein 1.2	GLFDIIKKIAESF-NH_2_	1479.76	0.582	0.765	+1
IK-1	GLFD**IIKK**IIKKIAESF-NH_2_	1962.42	0.540	0.780	+3
IK-2	GLFD**IIKKIIKK**IIKKIAESF-NH_2_	2445	0.514	0.714	+5
IK-3	GLFD**IIKKIIKK**IIKKI-NH_2_	2010.64	0.552	0.798	+6
KLA-1	GLFDIIKK**L**AESF-NH_2_	1479.76	0.574	0.759	+1
KLA-2	GLFDIIKK**L**A**KLA**ESF-NH_2_	1792	0.530	0.670	+2

**Table 2:** The MIC and MBC values of aurein 1.2 and its analogues.

Microorganism		Aurein 1.2	IK-1	IK-2	IK-3	KLA-1	KLA-2
		MIC/MBC (μM)					
Gram-positive bacteria	*S. aureus* (ATCC CRM 6538)	8/8	8/8	>128	16/16	16/16	2/2
	MRSA (ATCC 12493)	16/16	16/16	>128	16/32	16/32	4/4
	*E. faecalis* (NCTC 12697)	32/32	8/16	>128	16/32	32/32	16/16
Gram-negative bacteria	*E. coli* (NCTC 8739)	16/16	8/8	8/8	4/4	8/16	2/2
	*P. aeruginosa* (ATCC 9027)	64/128	16/32	64/128	16/16	64/128	16/16
	*K. pneumoniae* (ATCC 43816)	64/64	>128	16/16	8/8	32/32	4/4

**Table 3:** The MBIC and MBEC values of all tested peptides.

Microorganisms	Aurein 1.2	IK-1	IK-2	IK-3	KLA-1	KLA-2
	MBIC/MBEC (μM)					
*S. aureus* (ATCC CRM 6538)	16/64	16/>128	>128/>128	16/>128	16/64	2/64
MRSA (ATCC 12493)	16/64	16/>128	>128/>128	32/>128	16/128	4/128
*E. faecalis* (NCTC 12697)	32/>128	16/>128	>128/>128	16/>128	32/>128	16/>128
*E. coli* (NCTC 8739)	64/64	64/64	>128/>128	32/32	64/128	16/32
*P. aeruginosa* (ATCC 9027)	64/>128	32/>128	>128/>128	32/>128	64/>128	16/>128
*K. pneumoniae* (ATCC 43816)	128/>128	>128/>128	>128/>128	>128/>128	128/>128	64/>128

**Table 4:** The calculated IC_50_ values (μM) of aurein 1.2 and its analogs.

IC_50_ (μM)	H838	U251MG	MCF-7
Aurein1-2	26.9 ± 0.80	38.4 ± 0.24	25.9 ± 0.79
IK-1	3.5 ± 0.74	3.4 ± 0.71	3.0 ± 0.76
IK-2	3.7 ± 0.77	3.6 ± 0.18	3.2 ± 0.87
IK-3	3.0 ± 0.74	3.3 ± 0.63	3.4 ± 0.71
KLA-1	29.4 ± 0.22	34.1 ± 0.21	17.6 ± 0.79
KLA-2	4.6 ± 0.12	5.0 ± 0.12	3.6 ± 0.79

**Table 5:** The hemolysis percentages of aurein 1.2 and its analogs at its MIC value for *E. coli*.

	MIC of *E. coli* (μM)	Hemolysis (%)
Aurein1-2	16	20.56
IK-1	8	58.11
IK-2	8	53.56
IK-3	4	15.51
KLA-1	8	0.11
KLA-2	2	3.67

## Data Availability

Not applicable.

## Supplementary Materials

The following supporting information can be downloaded at: https://www.mdpi.com/article/10.3390/antibiotics12020412/s1, Figure S1: The RP-HPLC chromatograms of purified (A) aurein 1.2, (B) IK-1, (C) IK-2, (D) IK-3, (E) KLA-1, and (F) KLA-2 monitored at 214 nm.
