# Supplementary material for: A Novel Strategy for the Design of Aurein 1.2 Analogs with Enhanced Bioactivities by Conjunction of Cell-Penetrating Regions
Source: Antibiotics (Basel). 2023 Feb 19;12(2):412. doi: 10.3390/antibiotics12020412 (PMC9952496; doi:10.3390/antibiotics12020412)
Supplement: Supplementary file 1 [file antibiotics-12-00412-s001.zip › antibiotics-2205983-supplementary.pdf]

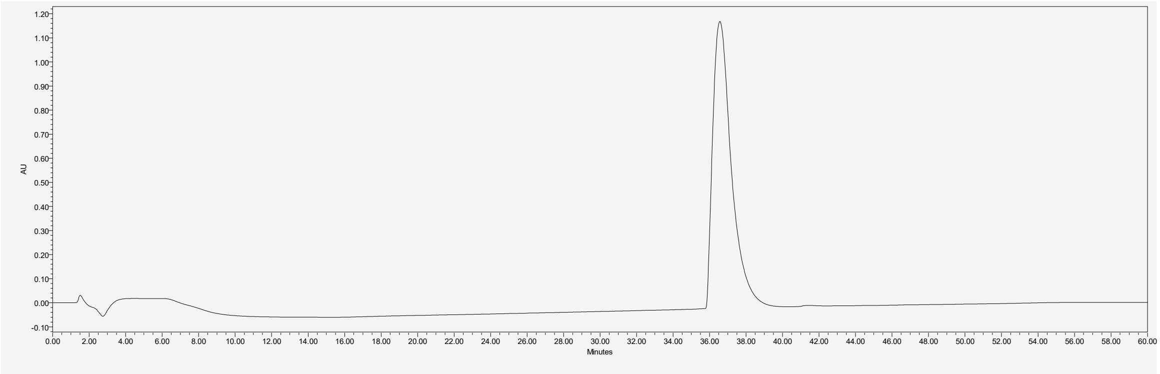
AU
1.20
1.10
1.00
0.90
0.80
0.70
0.60
0.50
0.40
0.30
0.20
0.10
0.00
-0.10
0.00 2.00 4.00 6.00 8.00 10.00 12.00 14.00 16.00 18.00 20.00 22.00 24.00 26.00 28.00 30.00 32.00 34.00 36.00 38.00 40.00 42.00 44.00 46.00 48.00 50.00 52.00 54.00 56.00 58.00 60.00
Minutes

(A)

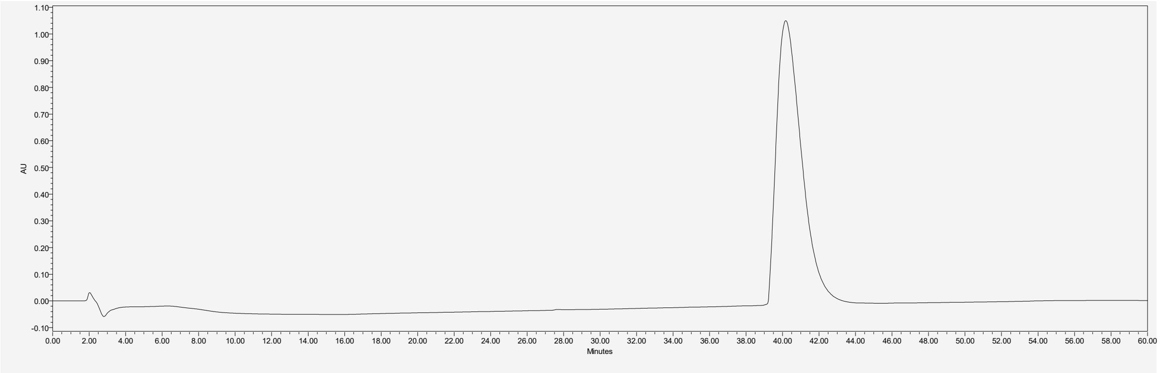
AU
1.10
1.00
0.90
0.80
0.70
0.60
0.50
0.40
0.30
0.20
0.10
0.00
-0.10
0.00 2.00 4.00 6.00 8.00 10.00 12.00 14.00 16.00 18.00 20.00 22.00 24.00 26.00 28.00 30.00 32.00 34.00 36.00 38.00 40.00 42.00 44.00 46.00 48.00 50.00 52.00 54.00 56.00 58.00 60.00
Minutes

(B)

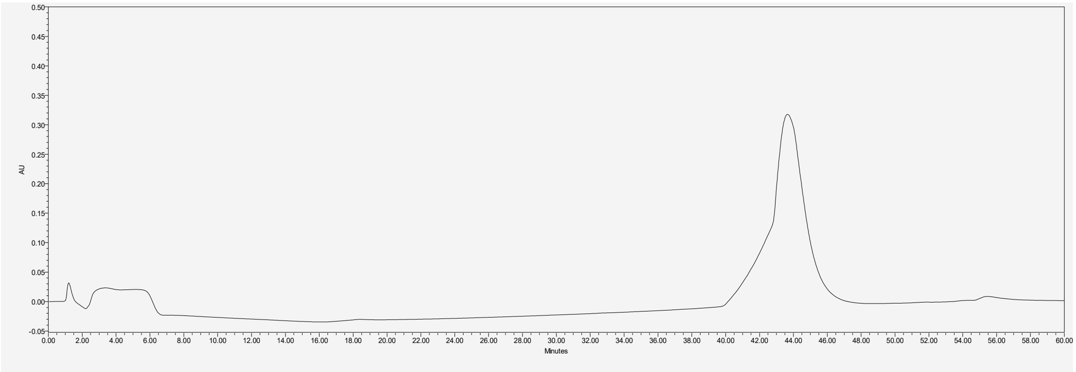
AU
0.50
0.45
0.40
0.35
0.30
0.25
0.20
0.15
0.10
0.05
0.00
-0.05
0.00 2.00 4.00 6.00 8.00 10.00 12.00 14.00 16.00 18.00 20.00 22.00 24.00 26.00 28.00 30.00 32.00 34.00 36.00 38.00 40.00 42.00 44.00 46.00 48.00 50.00 52.00 54.00 56.00 58.00 60.00
Minutes

(C)

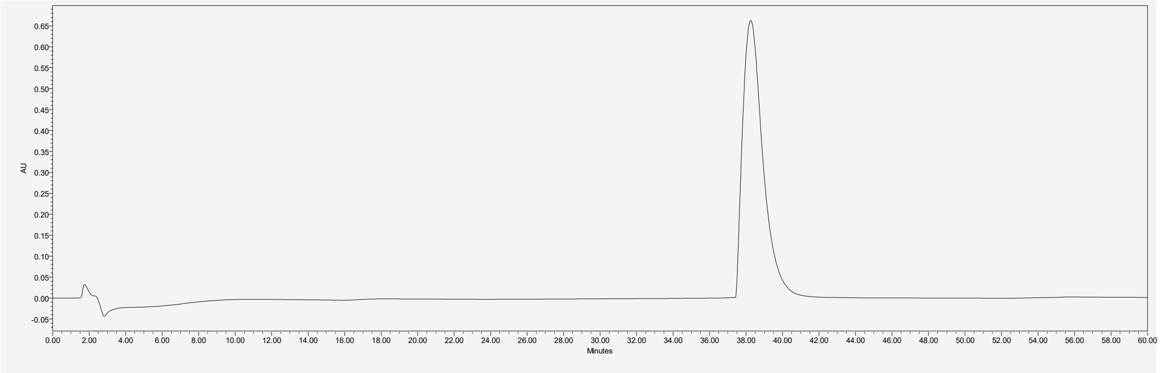

(D)

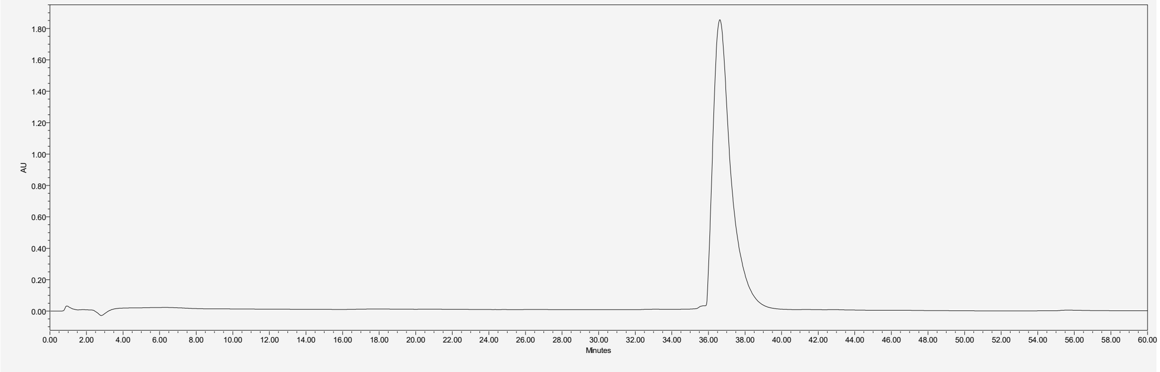

(E)

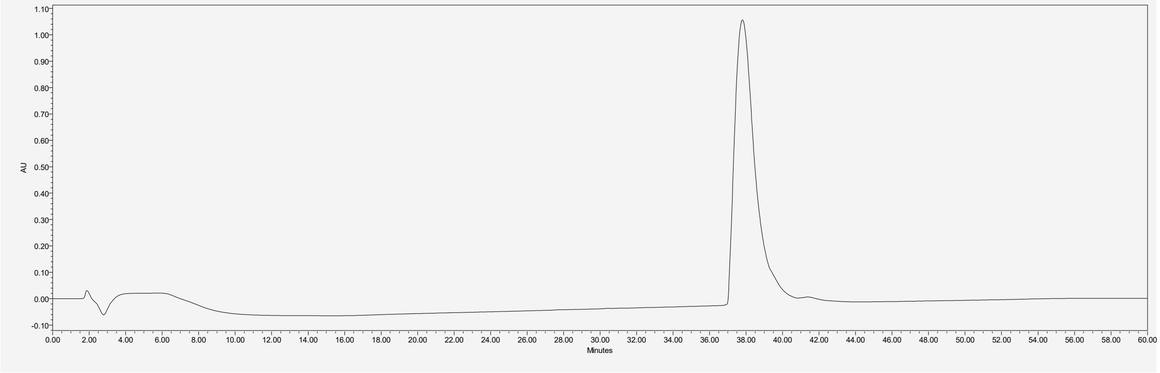

(F)

Figure S1. The RP-HPLC chromatograms of purified (A) aurein 1.2, (B) IK-1, (C) IK-2, (D) IK-3, (E) KLA-1 and (F) KLA-2 monitored at 214 nm.

.
